# Apixaban in Abdominal Transplantation: An Antithrombotic Stewardship Assessment

**DOI:** 10.1155/ah/5894289

**Published:** 2026-08-25

**Authors:** Diana Kwiatkowski, Tania Ahuja, Roshani Patolia, Dawn M. Pluckrose, David Green, Srijana Jonchhe

**Affiliations:** ^1^ Department of Pharmacy, NYU Langone Health, 550 First Avenue New York, New York 10016, USA, nyulangone.org; ^2^ Department of Medicine, NYU Grossman School of Medicine, 550 First Avenue, New York, New York 10016, USA, duhs.edu.pk

**Keywords:** apixaban, DOAC, solid organ transplant, SOT

## Abstract

**Background:**

Apixaban is a direct oral anticoagulant (DOAC) that is widely used in the general population; however, its use in solid organ transplant (SOT) recipients has not been extensively studied. Although there is interest in DOACs as the preferred oral anticoagulants in SOT recipients due to their favorable pharmacokinetic properties, there remain concerns for the optimal dose in the setting of organ dysfunction, at the time of engraftment, and for use for off‐label indications such as graft thrombosis (GT).

**Objective:**

To assess “real‐world” apixaban utilization in abdominal SOT recipients at a large academic medical center.

**Design:**

This was a single‐center, observational, retrospective cohort study of 79 adult patients from January 2020 to August 2022 who received apixaban for any indication postabdominal transplant.

**Methods:**

All adult patients, ≥ 18 years of age, who received apixaban for any indication post kidney, liver, simultaneous liver–kidney (SLK), or simultaneous pancreas–kidney (SPK) transplant were included. Electronic health records were retrospectively reviewed for patient demographics, past medical history, and apixaban characteristics, including indication, dose, frequency, and the use of parenteral anticoagulation lead‐in for acute thrombosis. The primary outcome was the incidence of thromboembolic events. Secondary outcomes included any bleeding events. In addition, adherence to package label dosing recommendations, based on indication of anticoagulation, was evaluated.

**Results:**

Among the 79 patients included, 51 (65%) were kidney, 18 (23%) were liver, 7 (9%) were SPK, and 3 (4%) were SLK transplant recipients. The most common indications for apixaban use were acute venous thromboembolism (VTE) (*n* = 33, 42%), stroke prevention with atrial fibrillation (AF) (*n* = 18, 23%), and treatment of GT (*n* = 13, 17%). The primary outcome of the incidence of any thromboembolic event was 3.8% (*n* = 3), which occurred at a median of 304 (IQR 168–309) days after apixaban initiation.

**Conclusions:**

Apixaban dosing was consistent with labeled dosing for the majority of the cohort for labeled indications, and the observed incidence of thromboembolic events in this cohort was low, with no excess identified relative to published literature. Our data support the use of apixaban for stroke prevention in AF or treatment of VTE or GT in patients who have received an abdominal transplant; however, risk factors for bleeding events, specifically in those with GT, should be further explored.

## 1. Introduction

Abdominal transplant recipients are at risk of developing postoperative thromboembolic complications, which can include venous thromboembolism (VTE), graft thrombosis (GT), and/or atrial fibrillation (AF) [1, 2]. Graft failure secondary to GT is more prevalent in pancreatic transplant recipients, with an incidence of 10%–35% compared to an incidence of 2%–9% in liver transplant recipients and 0.1%–4.2% in kidney transplant recipients [3, 4]. Transplant recipients have been described as being in a hypercoagulable state after organ transplant, especially during the first 6 months posttransplant, which is evident by the higher incidence of VTE seen in transplant recipients at 2%–14% for kidney transplant and 3%–5% for liver transplant, compared to the general population at 0.1%–0.2% [5]. Renal allograft thrombosis, either arterial or venous, is expected to occur in 1%–6% of cases [6]. The incidence of new‐onset AF has been reported to be 7.3% for kidney transplant recipients and 8.5% for liver transplant recipients, further predisposing these organ transplant recipients to other vascular complications such as stroke [7, 8].

Direct oral anticoagulants (DOACs), including apixaban, rivaroxaban, edoxaban, and dabigatran, have become widely utilized over warfarin, a vitamin K antagonist, due to the lack of necessary therapeutic drug monitoring and fewer drug–drug and drug–food interactions [9]. Additionally, the 2016 CHEST guidelines recommend DOACs over warfarin for the treatment of VTE due to reduced all‐cause mortality, fewer recurrent VTEs, and less major bleeding when compared to low molecular weight heparin and warfarin [10]. While DOACs are the preferred anticoagulants in the general population, the data examining safety and efficacy in solid organ transplant (SOT) recipients is limited, as this population was not included in DOAC landmark trials [11]. Consequently, there are many unanswered questions revolving around the use of DOACs in this special population, such as the impact of drug–drug interactions (DDIs) with DOACs and immunosuppressants and prophylactic anti‐infectives and the optimal dose and duration of anticoagulation for GT [12]. Due to the risks of thromboembolic events and cardiovascular complications associated with abdominal transplantation, standardization of appropriate dosing, timing of DOAC initiation posttransplantation, and assessment of clinically significant DDIs is necessary.

There are several factors that may limit the use of DOACs in abdominal transplant recipients and may prompt off‐label dose adjustments to be made. Elevated serum creatinine (SCr) concentrations and fluctuating graft function may cause hesitancy in initiating DOACs shortly after transplantation in kidney transplant recipients, as these agents are hepatically metabolized and renally eliminated, with dabigatran renal elimination at 85%, edoxaban at 35%–50%, rivaroxaban at 36%, and apixaban at 25% [9, 10]. Moreover, certain peri‐transplant medications used for induction immunosuppression and opportunistic infection prophylaxis may impact hemostatic parameters, leading to thrombocytopenia, or can reduce the metabolism of DOACs, predisposing transplant recipients to potential bleeding risks. Managing DOACs in patients who are undergoing an unplanned transplant surgery or urgent allograft biopsy can also prove to be challenging [13]. Weighing the aforementioned risks and benefits, the abdominal transplant team at NYU Langone Health (NYULH), a large academic medical center, has preferentially chosen DOACs, specifically apixaban, in patients requiring treatment of VTE or stroke prevention in AF. The transplant pharmacists serve the role of antithrombotic stewards to review, assess, recommend, and monitor all anticoagulants used in patients receiving an abdominal transplant. Thus, the purpose of this study is to characterize apixaban prescribing patterns in patients who have received either a kidney, liver, or pancreas transplant and assess clinical outcomes, including the incidence of thromboembolic events and/or bleeding events. We hypothesized that apixaban use in abdominal transplant patients would most commonly be used for treatment of VTE or stroke reduction with AF, and that there would be heterogeneity in dosing recommendations, taking concomitant medications and time from transplant into account.

## 2. Methods

### 2.1. Study Population and Outcomes

This was an observational, single‐center, retrospective cohort study of apixaban utilization at NYULH, a 925‐bed tertiary academic medical center, from January 2020 to August 2022. Our hospital is part of a large health system; however, all abdominal transplants occur at our site. All adult patients, ≥ 18 years of age, who received apixaban for any indication post kidney, liver, simultaneous liver–kidney (SLK), or simultaneous pancreas–kidney (SPK) transplant at NYULH in the inpatient setting were included. Any patient who received an abdominal transplant and did not receive at least 2 doses of apixaban or received any other anticoagulant was excluded. Given the retrospective nature of this observational review for quality assessment purposes, informed consent was not required. This study was conducted as a quality improvement initiative approved by NYULH’s transplant program and was determined not to constitute human subjects research; as such, formal Institutional Review Board approval was not required. All patient data were de‐identified prior to analysis. The primary outcome was the incidence of any thromboembolic event after apixaban initiation. Thromboembolic events included any stroke, pulmonary embolism (PE), deep vein thrombosis (DVT), or GT, as documented in the electronic health record. Diagnostic criteria for recurrent PE included a computerized tomography (CT) scan or pulmonary angiography‐confirmed diagnosis of PE or a new PE confirmed at autopsy. Diagnostic criteria for recurrent DVT included a new noncompressible venous segment or a substantial increase in the diameter of the thrombus during full compression in a previously abnormal segment on ultrasonography or determined on venography. The secondary outcome was the incidence of bleeding within 3 months after apixaban initiation, as documented in the electronic health record. Major bleeding and clinically relevant non‐major bleeding (CRNMB) events were defined according to the International Society on Thrombosis and Haemostasis (ISTH). In addition, adherence to package label dosing recommendations, based on indication of anticoagulation, was evaluated. For nonvalvular AF, standard dosing per label is apixaban 5 mg twice daily, with dose reduction to 2.5 mg twice daily required in patients meeting at least 2 of the following 3 criteria: age ≥ 80 years, body weight ≤ 60 kg, or SCr ≥ 1.5 mg/dL. For treatment of VTE (DVT/PE), label dosing is apixaban 10 mg twice daily for 7 days, followed by 5 mg twice daily; for reduction in risk of recurrent DVT/PE after at least 6 months of treatment, the label dose is 2.5 mg twice daily. For dosing for off‐label indications, dosing was recorded but not assessed for appropriateness. Exploratory analysis included characteristics of bleeding events, including the incidence of major and minor bleed events, use of apixaban and parenteral “lead‐in” dosing prior to bleed events, and transplant type in patients who experienced a bleed event.

### 2.2. Study Definitions

Concomitant DDIs were considered if patients received medications that could impact apixaban metabolism or increase the risk of bleeding when used concurrently with apixaban. These included reviewing for concomitant CYP3A4 inhibitors, including cobicistat, itraconazole, ketoconazole, posaconazole, ritonavir, amiodarone, diltiazem, dronedarone, verapamil, or voriconazole. In addition, concomitant CYP3A4 inducers, including carbamazepine, rifampin, oxcarbazepine, phenytoin, phenobarbital, bosentan, and primidone, were reviewed. A full lead‐in dosing scheme was defined as receipt of 7 days of apixaban 10 mg twice daily or parenteral anticoagulation (i.e., heparin continuous infusion with anti‐Xa monitoring). A reduced lead‐in dosing scheme was defined as receipt of less than 7 days of apixaban 10 mg twice daily, or no lead‐in altogether [14, 15]. Major bleeds were defined consistent with ISTH definitions, where bleeds causing a decrease in hemoglobin of ≥ 2 g/dL or receipt of two or more units of packed red blood cells were considered major bleeds [16]. Creatinine clearance was calculated using the Cockcroft–Gault equation.

### 2.3. Data Collection and Statistics

Electronic health records were retrospectively reviewed for patient demographics, past medical history, and apixaban characteristics, including indication, dose, frequency, and the use of parenteral anticoagulation lead‐in for acute thrombosis. Additional data collected included transplant type, indication for transplant, concomitant medications utilized, thromboembolic events, and bleeding events. All data collected were managed utilizing Research Electronic Data Capture (REDCap), which is a secure informatics system designed to support data collection across various research disciplines [17, 18]. Categorical variables were described as frequencies with proportions. Continuous variables were described as medians with interquartile ranges (IQRs) and analyzed using the Mann–Whitney *U* test. All data were secured, and statistics were conducted using SPSS Statistics Software Version 28 (IBM Corp, Armonk, NY) [19].

## 3. Results

Among the 79 patients included, 51 (65%) were kidney, 18 (23%) were liver, 7 (9%) were SPK, and 3 (4%) were SLK transplant recipients (Table 1). The majority of the cohort was male (66%), with a median age of 55 (IQR 48–67) years and a median baseline creatinine clearance of 45 (IQR 22–65) mL/min at the time of apixaban initiation. Hypertension was the most common comorbidity at 75% (*n* = 59), followed by diabetes mellitus at 43% (*n* = 32), AF at 23% (*n* = 18), cancer at 16% (*n* = 13), history of cerebrovascular accident at 11% (*n* = 9), and history of VTE in 10% (*n* = 8). Apixaban was utilized in 20% of the cohort prior to abdominal transplant. Concomitant medications that may increase the risk of bleeding while on apixaban, including immunosuppression or antiviral therapy, which may portend thrombocytopenia, and CYP3A4 inhibitors, were recorded, with 85% of the cohort receiving tacrolimus (*n* = 67), 75% of the cohort receiving valganciclovir (*n* = 59), and 23% of the population receiving fluconazole (*n* = 18). Other concomitant medications can be found in Table 1. Apixaban use, by transplant type, is detailed in Table 2.

**TABLE 1:** Baseline characteristics.

Characteristic	*N* = 79
Sex, male; *n* (%)	52 (66)
Age, years; median (IQR)	55 (48–67)
Weight, kg; median (IQR)	74.4 (64.3–94.6)
CrCl, mL/min; median (IQR)	44.6 (22.1–64.9)
Past medical history, *n* (%)	
Hypertension	59 (75)
Diabetes mellitus	34 (43)
Coronary artery disease	20 (25)
Atrial fibrillation	18 (23)
Cirrhosis	17 (22)
Cancer	13 (16)
Heart failure	13 (16)
Peripheral vascular disease	12 (15)
Cerebrovascular accident	9 (11)
Venous thromboembolism	8 (10)
Clotting disorder	4 (5)
Transplant type, *n* (%)	
Kidney	51 (65)
Liver	18 (23)
SPK	7 (9)
SLK	3 (4)
Concomitant medications, *n* (%)	
Transplant medications	
Tacrolimus	67 (85)
Belatacept	12 (15)
Valganciclovir	59 (75)
Sulfamethoxazole–trimethoprim	52 (66)
Cyclosporine	0 (0)
CYP3A4 inhibitors and antiplatelets	
Aspirin	25 (32)
Fluconazole	18 (23)
Amiodarone	5 (6)
Isavuconazole	1 (1)

*Note:* CrCl: creatinine clearance; SPK: simultaneous pancreas–kidney transplant; SLK: simultaneous liver–kidney transplant.

**TABLE 2:** Apixaban use by transplant type.

	Total, *N* = 79	KT, *n* = 51 (65)	LT, *n* = 18 (23)	SPK, *n* = 7 (9)	SLK, *n* = 3 (4)
Age, years; median (IQR)	55 (48–67)	60 (49–68)	55 (48–64)	50 (42–50)	56 (53–60)
Weight, kg; median (IQR)	74.4 (64.3–94.6)	76.1 (67.1–100.2)	71.3 (57.7–87.8)	73 (64.3–96.9)	68.1 (63.2–70.7)
Serum creatinine, mg/dL; median (IQR)	1.77 (1.24–3.29)	2.4 (1.71–5.11)	0.9 (0.73–1.32)	1.33 (0.99–1.57)	0.88 (0.81–1.18)
CrCl, mL/min; median (IQR)	45 (22–65)	27 (12–52)	73 (61–96)	69 (53–76)	85 (60–88)
Initiation posttransplant, days; median (IQR)	21 (9–79)	19 (7–109)	27 (11–39)	23 (7–248)	57 (54–475)
Apixaban indication, *n* (%)					
Acute VTE	33 (42)	21 (41)	9 (50)	1 (14)	2 (67)
AF, stroke prevention	18 (23)	18 (35)	0	0	0
GT	13 (16)	0	6 (33)	6 (86)	1 (33)
Other	15 (19)	12 (24)	3 (17)	0	0
Apixaban dosing, *n* (%)					
5 mg BID	68 (86)	41 (80)	17 (94)	7 (100)	3 (100)
2.5 mg BID	11 (14)	10 (20)	1 (6)	0	0
Apixaban lead‐in dose for VTE and GT indication, *n* (%)	20/46 (43)	4/21 (19)	5/15 (33)	0	1 (33)
Full	10 (50)	2 (50)	2 (40)	0	0
Partial	10 (50)	2 (50)	3 (60)	0	1 (100)

*Note:* SPK: simultaneous pancreas–kidney transplant; SLK: simultaneous liver–kidney transplant; CrCl: creatinine clearance; VTE: venous thromboembolism.

Abbreviations: AF, atrial fibrillation; GT, graft thrombosis; KT, kidney transplant; LT, liver transplant.

The primary outcome of the incidence of any thromboembolic event in our cohort was 3.8% (*n* = 3; 95% CI, 0.8%–10.7%), which occurred at a median of 304 (IQR 168–309) days after apixaban initiation. All three events were DVTs. Two events occurred in patients initiated on anticoagulation for stroke prevention in AF, and one event was a recurrent DVT that occurred in a patient on apixaban for treatment of VTE. Apixaban dosing for stroke prevention with AF was consistent with FDA‐approved dosing for most patients. In this cohort, patients receiving apixaban for AF who were dose‐reduced had a median SCr greater than 1.5 mg/dL, therefore only meeting one out of three criteria for apixaban dose reduction for AF (Table 2). Sixty percent of abdominal transplant recipients received a partial or full lead‐in, with either apixaban or parenteral therapy for VTE indications. While not all patients in the cohort received lead‐in therapy, the overall incidence of thromboembolism was low (Table 2). The maintenance dosing for VTE was consistent with FDA‐labeled dosing for most of the cohort, approximately 88% of the VTE population. No thromboembolic events occurred in recipients who received a reduced maintenance dose (Table 2). Although apixaban is not FDA‐approved for the treatment of GT, all patients with this indication received apixaban 5 mg twice daily, and one of these patients also received apixaban lead‐in therapy. The incidence of bleeding events in this cohort was 11.4% (*n* = 9; 95% CI, 5.3%–20.5%), with six out of nine considered major bleeds. Bleed events occurred at a median of 32 (IQR 13–63) days after apixaban initiation (Table 3). Of the nine patients with bleeding events, five were prescribed aspirin, and two were prescribed fluconazole at the time of their bleeding event. Despite concerns for DDIs associated with apixaban and commonly prescribed posttransplant medications, we did not identify use of these agents as a risk for empiric dose reduction in most patients (Table 3). Apixaban was continued in 5 out of the 9 bleeds. With regard to the 4 discontinuations, 1 was discontinued for vaginal bleeding in a patient on apixaban for > 1 year, where the decision for extended therapy was not necessary. There were 2 patients with gastrointestinal bleeds, and the risk–benefit analysis tipped toward stopping apixaban; for 1 discontinuation, it was used off‐label, but there was a pseudoaneurysm of the iliac graft, and so it was discontinued. No major reoperations were required for any of the bleeds.

**TABLE 3:** Bleeding events by patient.

Case	Severity	Bleed location	SOT type	Apixaban initiation post‐SOT (days)	Indication	Dose	Time to bleed from apixaban initiation (days)	SCr (mg/dL)	DDIs	Apixaban discontinued
1	Major	Vaginal	Kidney	6	Prior DVT	2.5 mg BID	306	0.81	N/A	Yes
2	Major	GI	Liver	10	GT	5 mg BID	104	0.91	Aspirin	No
3	Major	GI	Kidney	146	VTE	5 mg BID	48	3.95	Aspirin	No
4	Major	Vaginal/pseudo‐aneurysm of donor iliac Y graft	SPK	32	GT	5 mg BID	12	1.28	Aspirin	Yes
5	Major	GI	Kidney	4	AF	5 mg BID	61	0.96	N/A	No
6	Major	GI	Liver	33	GT	5 mg BID	14	1.21	Aspirin, fluconazole	Yes
7	Minor	GI	Liver	27	VTE	2.5 mg BID	7	1.98	Aspirin, fluconazole	Yes
8	Minor	Retroperitoneal hematoma	Kidney	7	AF	2.5 mg BID	7	4.72	N/A	No
9	Minor	GI	Kidney	5	VTE	5 mg BID	16	3.36	N/A	No

*Note:* GI: gastrointestinal; VTE: venous thromboembolism; BID: twice daily.

Abbreviations: AF, atrial fibrillation; DVT, deep vein thrombosis; GT, graft thrombosis; N/A, not applicable; SOT, solid organ transplant.

## 4. Discussion

Our study demonstrates the safe use of apixaban in patients who received either a kidney, liver, or pancreas transplant at a large academic medical center. We observed a low incidence of thromboembolic events when apixaban was used for any indication in our heterogeneous abdominal SOT cohort, consistent with recurrent thromboembolic events observed in the landmark trials of apixaban for stroke prevention with AF and for the treatment of VTE, though lower than those observed in those with VTE and cancer [20–24]. Two VTE events occurred in patients who were initially prescribed apixaban for stroke prevention with AF, and one VTE event occurred in a patient with high risk of recurrence due to prior recurrent VTEs. As these VTEs occurred at a median of 304 days after apixaban initiation, we believe these events may have occurred due to underlying thromboembolic risk factors that we did not account for in our retrospective review. Further, although there were deviations from labeled dosing regarding initial lead‐in therapy with apixaban 10 mg twice daily or parenteral anticoagulation and dose reductions when apixaban was prescribed for stroke prevention with AF, the three thromboembolic events did not occur in patients who received these off‐label doses. Treatment of VTE in kidney transplant recipients can be a challenge given dynamic renal function, and thus prescribing patterns may differ from labeled dosing in an attempt to mitigate bleeding risks. This was observed in our cohort, with clinicians opting out of the 10 mg lead‐in for VTE or with dose reductions occurring in those with an SCr > 2.5 mg/dL. Although we do not advocate for off‐label use in the absence of a randomized controlled trial, our cohort demonstrates real‐world prescribing patterns and highlights which patient populations resulted in deviations, such as those with GT. Given the retrospective nature of our study, we cannot ascertain all the decisions that resulted in off‐label dose reductions. However, DOAC use should generally be avoided in the immediate postoperative period and should only be considered after stability of renal and hepatic function has been established and when bleeding risk has stabilized. It is possible that the transplant team’s perception of bleeding events was high in relation to time from transplant and perceived risk of DDIs that led to empiric dose reductions or off‐label use in our cohort.

Our study is unique in that we observed the use of apixaban for atypical thrombus locations, including the treatment of GT. Clinical practice for anticoagulation strategies for the prevention and treatment of GT is currently based mostly on retrospective cohorts and small‐sample‐sized randomized clinical trials, leading to heterogeneous practice amongst different institutions, with diverse anticoagulation protocols [25, 26]. As there are no randomized data evaluating anticoagulation strategies of GT, there remains equipoise surrounding the optimal anticoagulation agent and dosing strategy. For patients in our cohort, when apixaban was used for the treatment of GT, we observed heterogeneity with the use of a full or partial lead‐in with either apixaban or parenteral AC. Although we cannot say for certain why these strategies were chosen, we believe that this mixed strategy may have been chosen due to the need for AC, without data suggesting a preference. Additionally, parenteral therapy may have been chosen for shorter drug half‐life properties in the setting of potential need for a procedure and/or used in patients hospitalized up‐front, with a plan to transition to oral apixaban therapy. As none of these patients had a recurrent thromboembolic event or thrombosed their graft, we believe a strategy of some lead‐in therapy followed by apixaban 5 mg twice daily may be reasonable for this patient population until more data becomes available. This is similar to a strategy that has been chosen for the treatment of thrombus in atypical locations, including intracardiac thrombosis or splanchnic vein thrombosis [3–5, 27–29].

In addition to preventing thromboembolic events, the incidence of bleeding events with anticoagulation in patients who receive an abdominal transplant must be balanced. We observed a bleed event rate of 11.4%, higher than the bleeding incidence reported in the landmark trials for apixaban, including in those with cancer; however, the major bleeding event rate in our cohort is consistent with bleed events reported in real‐world assessments of apixaban use in special populations [12, 20–24]. In a study of 106 SOT recipients (70 on apixaban, 36 on rivaroxaban or dabigatran), the cumulative incidence of any bleeding was significantly lower in the apixaban group at both 90 days (4.9% vs. 16.1%) and 180 days (11.4% vs. 24.9%, *p* = 0.034). Rates of major bleeding and thrombosis were similar between groups [30]. Most of our bleeding events occurred within a month of apixaban initiation, although two occurred greater than 3 months after initiation. Surprisingly, the SCr at the time of the bleeding event was > 1.5 mg/dL in only four of the nine bleeds. In a national survey among transplant pharmacists in the US in 2019, DOAC use was permissive in the posttransplant setting by 94.3% of the responding programs; however, patients’ renal function and concomitant DDIs were among major factors that influenced prescribing decisions [10]. Since there remain concerns about using apixaban in the setting of DDIs, including the use of medications that may lead to thrombocytopenia, potentiating the risk for bleeding, we evaluated concomitant interacting medications to identify potential risk factors for bleeding. Despite theoretical concerns about DDIs with calcineurin inhibitors, elevated apixaban exposure after coadministration with cyclosporine is not anticipated to pose clinically relevant bleeding events based on safety analyses. In the case of tacrolimus, a modest reduction in apixaban exposure is not expected to result in meaningful loss of efficacy [31]. Notably, we did identify the use of aspirin along with apixaban in 5 of the 9 bleeding events. The use of antiplatelet therapy plus anticoagulation is known to be associated with an increased risk of bleeding, with dual antiplatelet therapy associated with the highest risk. However, even single antiplatelet therapy with anticoagulation portends a greater bleeding risk than anticoagulation therapy alone [32–34]. Thus, antithrombotic stewardship is essential to evaluate the entire antithrombotic regimen and assess if patients can discontinue single antiplatelet therapy while on anticoagulation therapy. This has to be balanced with the ischemic risk, particularly in liver transplantation, where aspirin therapy is commonly used for the prevention of early hepatic artery thrombosis, a critical postoperative complication that can lead to graft loss [35]. Furthermore, testing of apixaban activity levels may be considered for select patients, especially in the setting of relevant DDIs, where the expected therapeutic dose–response of apixaban may be less defined. Transplant patients frequently receive azole antifungals (voriconazole, fluconazole, and posaconazole) for prophylaxis or treatment. These are potent dual inhibitors of CYP3A4 and permeability glycoprotein (P‐gp), and their concomitant use with apixaban can substantially increase drug exposure and bleeding risk. Our cohort did consist of 2 patients receiving concomitant fluconazole at the time of the bleeding event, but other antifungal use was not prevalent. Our cohort did not consist of any apixaban‐calibrated anti‐factor Xa testing or drug‐specific levels to assess this dose–response, but this may be considered for other SOTs, with DDIs or in the setting of bleeding events.

Our study demonstrates real‐world prescribing of apixaban for abdominal transplant recipients. Overall, we observed apixaban to be well tolerated, though bleed events were more common when apixaban was used for the treatment of GT. Our study is limited by its retrospective design and modest sample size. Data were collected from existing medical records rather than through prospective, protocolized data collection, which may have led to documentation bias. The outcomes and clinical decisions that were not formally recorded could not be captured or analyzed, including the use of central venous catheters and/or anti‐T cell induction of treatment or rejection, which may all portend increased thromboembolic risk. Unlike a randomized controlled trial, there was no ability to control for confounding variables at the time of prescribing, limiting causal inference. All conclusions are therefore associative rather than definitively causal. Prescribing decisions were made at the discretion of individual clinicians, meaning that channeling bias is likely present, where the transplant teams may have preferentially chosen apixaban for patients perceived to be at lower bleeding risk or with favorable renal and hepatic profiles. Furthermore, our findings may not be generalizable to other transplant programs, which may differ in their immunosuppression protocols, patient demographics, prescribing culture, and access to resources. Practice patterns at a large academic transplant center may differ substantially from community settings. In addition, we may have observed heterogeneity by pooling kidney, liver, and pancreas recipients, given variability in baseline coagulation status, underlying comorbidities, posttransplant complications, and organ‐specific pharmacokinetic considerations. This heterogeneity may obscure organ‐specific prescribing patterns or outcomes and limit the applicability of findings to any single transplant type. In addition, we were unable to ascertain the reasons for all dose reductions in our cohort; though we hypothesized why they may have occurred, this variability limits the ability to draw conclusions about the optimal dosing strategy in this patient population. Without apixaban drug level monitoring (anti‐factor Xa activity) or pharmacokinetic sampling, it is impossible to determine whether prescribed doses resulted in therapeutic, sub‐therapeutic, or supratherapeutic drug exposure in individual patients. This is a particularly important limitation given the known pharmacokinetic variability introduced by CNI coadministration and fluctuating renal function in this population. Lastly, we may have observed confounding bias by indication, as patients prescribed apixaban for stroke prevention due to AF may differ systematically from those prescribed it for VTE treatment or prophylaxis, with different underlying risk profiles or multivariable adjustment; confounding by indication may influence observed patterns. Despite these limitations, our study has many strengths. One of the most important strengths of this study is that it directly addresses a clinically meaningful and underexplored area. SOT recipients have historically been excluded from major DOAC clinical trials, meaning that real‐world prescribing data from this population are scarce. A study characterizing how apixaban is being used in clinical practice provides valuable information that cannot be obtained from pivotal trial data alone and contributes meaningfully to the limited literature guiding anticoagulation decisions in this complex population. The prescribing patterns captured represent the decisions made by experienced transplant clinicians managing real patients with the full complexity of posttransplant comorbidities, polypharmacy, and organ dysfunction. This enhances the external validity of the findings for practicing clinicians. Further, we have dedicated transplant pharmacists who are closely involved in anticoagulation management, lending credibility to the prescribing decisions captured and potentially reducing practice variation within the dataset. Even in the absence of a control arm, our study serves an important scientific function by identifying areas of practice variability, inconsistency in dose reduction criteria application, and gaps between guideline recommendations and clinical behavior. These findings directly inform the design of future prospective studies, quality improvement initiatives, and institutional protocol development.

In conclusion, our data support the use of apixaban for stroke prevention in AF or treatment of VTE or GT in patients who have received an abdominal transplant [36–38]. However, the higher incidence of bleeding events in GT should be further explored in larger cohorts to assess risk factors. Future prospective studies are required to confirm the safety and efficacy of DOACs and evaluate which DOAC may be preferred. The use of apixaban for stroke prevention in AF, treatment of VTE, or GT appears safe; however, risk factors for bleeding events, specifically in those with GT, should be further explored.

## Author Contributions

Diana Kwiatkowski, PharmD: conceptualization, data curation, formal analysis, investigation, methodology, writing–original draft, writing–reviewing and editing.

Tania Ahuja, PharmD: conceptualization, data curation, formal analysis, methodology, project administration, resources, supervision, validation, writing–original draft, writing–reviewing and editing.

Roshani Patolia, PharmD: conceptualization, data curation, formal analysis, methodology, project administration, resources, supervision, validation, writing–reviewing and editing.

Dawn M. Pluckrose, PharmD: conceptualization, data curation, formal analysis, methodology, supervision, validation, writing–reviewing and editing.

David Green, MD, PhD: methodology, writing–reviewing and editing.

Srijana Jonchhe: conceptualization, data curation, formal analysis, methodology, project administration, resources, supervision, validation, writing–original draft, writing–reviewing and editing.

## Funding

The authors received no financial support for the research, authorship, and/or publication of this article.

## Disclosure

All authors have read and approved the final version of the manuscript. Tania Ahuja had full access to all of the data in this study and takes complete responsibility for the integrity of the data and the accuracy of the data analysis. Preliminary data of these results were presented in poster form at the International Society of Thrombosis and Hemostasis (ISTH) Meeting in Montreal, CN, from June 24 to 28, 2023, with a corresponding abstract published: Kwiatkowski, D., Patolia, R., Pluckrose, D., Jonchhe, S., & Ahuja, T. (2023). PB0495 Apixaban Use in Abdominal Transplant Recipients at a Large Academic Medical Center. Research and Practice in Thrombosis and Haemostasis, 7, 101879.

## Ethics Statement

Given the retrospective nature of this observational review for quality assessment purposes, informed consent was not required. All patient data were de‐identified and collected in compliance with NYU Langone Health’s institutional review board exempt/waived protocols.

## Consent

Patient consent was exempt from the NYU Langone Health IRB due to the retrospective nature.

## Conflicts of Interest

The authors declare no conflicts of interest.

## Data Availability

The data that support the findings of this study are available from the corresponding author upon reasonable request.
